# Tabby2: a user-friendly web tool for forecasting state-level TB outcomes in the United States

**DOI:** 10.1186/s12916-023-02785-y

**Published:** 2023-08-30

**Authors:** Nicole A. Swartwood, Christian Testa, Ted Cohen, Suzanne M. Marks, Andrew N. Hill, Garrett Beeler Asay, Jennifer Cochran, Kevin Cranston, Liisa M. Randall, Andrew Tibbs, C. Robert Horsburgh, Joshua A. Salomon, Nicolas A. Menzies

**Affiliations:** 1 Department of Global Health and Population, Harvard T.H. Chan School of Public Health, Boston, MA 02120 USA; 2 Department of Social and Behavioral Sciences, Harvard T.H. Chan School of Public Health, Boston, MA USA; 3 Department of Epidemiology of Microbial Diseases, Yale School of Public Health, New Haven, CT USA; 4 Division of Tuberculosis Elimination, National Center for HIV, Viral Hepatitis, STD, and TB Prevention, Centers for Disease Control and Prevention, Atlanta, GA USA; 5 Bureau of Infectious Disease and Laboratory Sciences, Massachusetts Department of Public Health, Boston, MA USA; 6 Departments of Epidemiology, Biostatistics, Global Health and Medicine, Boston University Schools of Public Health and Medicine, Boston, MA USA; 7 Center for Health Policy / Center for Primary Care and Outcomes Research, Stanford University, Stanford, USA

**Keywords:** Tuberculosis, Infectious disease, Mathematical modeling, Web application, Epidemiology

## Abstract

**Background:**

In the United States, the tuberculosis (TB) disease burden and associated factors vary substantially across states. While public health agencies must choose how to deploy resources to combat TB and latent tuberculosis infection (LTBI), state-level modeling analyses to inform policy decisions have not been widely available.

**Methods:**

We developed a mathematical model of TB epidemiology linked to a web-based user interface — Tabby2. The model is calibrated to epidemiological and demographic data for the United States, each U.S. state, and the District of Columbia. Users can simulate pre-defined scenarios describing approaches to TB prevention and treatment or create their own intervention scenarios. Location-specific results for epidemiological outcomes, service utilization, costs, and cost-effectiveness are reported as downloadable tables and customizable visualizations. To demonstrate the tool’s functionality, we projected trends in TB outcomes without additional intervention for all 50 states and the District of Columbia. We further undertook a case study of expanded treatment of LTBI among non-U.S.–born individuals in Massachusetts, covering 10% of the target population annually over 2025-2029.

**Results:**

Between 2022 and 2050, TB incidence rates were projected to decline in all states and the District of Columbia. Incidence projections for the year 2050 ranged from 0.03 to 3.8 cases (median 0.95) per 100,000 persons. By 2050, we project that majority (> 50%) of TB will be diagnosed among non-U.S.–born persons in 46 states and the District of Columbia; per state percentages range from 17.4% to 96.7% (median 83.0%). In Massachusetts, expanded testing and treatment for LTBI in this population was projected to reduce cumulative TB cases between 2025 and 2050 by 6.3% and TB-related deaths by 8.4%, relative to base case projections. This intervention had an incremental cost-effectiveness ratio of $180,951 (2020 USD) per quality-adjusted life year gained from the societal perspective.

**Conclusions:**

Tabby2 allows users to estimate the costs, impact, and cost-effectiveness of different TB prevention approaches for multiple geographic areas in the United States. Expanded testing and treatment for LTBI could accelerate declines in TB incidence in the United States, as demonstrated in the Massachusetts case study.

**Supplementary Information:**

The online version contains supplementary material available at 10.1186/s12916-023-02785-y.

## Background

Approximately 9000 persons were diagnosed with tuberculosis (TB) disease yearly in the United States over 2017–2019. In these years, 12–13% of reported TB cases were attributed to recent transmission [1], with the remainder likely resulting from progression of latent tuberculosis infections (LTBI) acquired >2 years previously. Recent estimates suggest that between 3.1% and 5.0% of the U.S. population have LTBI [2, 3] which may progress to TB disease in the future. TB infection and disease are not equally distributed across U.S. populations; LTBI prevalence is higher among non-U.S.–born persons, likely exposed to TB prior to U.S. arrival, and U.S.–born persons exposed in congregate settings [4]. A small number of U.S. states—particularly those with large non-U.S.–born populations—report substantial numbers of TB cases: California, Florida, Texas, and New York collectively reported 50.6% of U.S. TB cases in 2017-2019 [1]. Annual reported TB cases dropped by 20% at the beginning of the COVID-19 pandemic in 2020 [5], with reduced immigration, reduced transmission, and interruptions in healthcare access each potentially playing a role. Prior to 2020, most states had reported modest annual declines in TB incidence, although several states (Alabama, Louisiana, Missouri, Minnesota, New Jersey, and South Carolina) reported greater numbers of TB cases in the period 2017–2019 compared to the preceding 3-year period [6]. Preliminary 2021 reported TB cases showed an increase compared to 2020, suggesting a return toward these pre-pandemic trends [7].

A major strategy for accelerating TB elimination in the United States is targeted testing and treatment (TTT) of populations at elevated risk of developing TB disease due to progression of LTBI. These populations include those with higher LTBI prevalence (non-U.S.–born individuals, persons experiencing homelessness, incarceration, or residing in other congregate settings) and individuals with elevated LTBI progression risks (individuals with immunosuppression, such as HIV, end-stage renal disease, or those taking an immunosuppressive therapy) [8]. In order for TB prevention programs to allocate resources effectively, it is critical for these programs to identify local populations at high risk for TB disease and design interventions which maximize the health impact of available funding for their jurisdiction. To support these goals, mathematical modeling can be used to synthesize epidemiological data and project future TB incidence and LTBI prevalence trends under a wide range of hypothetical future scenarios, while taking into account of local factors that will affect the costs and impact of proposed policies. Combined with cost-effectiveness analyses, modeling can identify intervention approaches that maximize the prevention impact for a given budget.

Here, we introduce Tabby2, a web application that allows users to conduct interactive epidemiological and economic analyses using a mathematical model of TB epidemiology in the United States. This model is calibrated to TB and demographic data for each U.S. state, the District of Columbia, and for the entire United States. Tabby2 allows users to select a geographic area, explore projections of TB outcomes under base case assumptions, specify intervention scenarios to be compared, and simulate a range of outcomes (future epidemiological trends, changes in health service utilization, costs, and cost-effectiveness) associated with each scenario. As an open-access web application, Tabby2 provides a user-friendly interface for use by researchers, health officials, and TB program staff to understand the implications of TB policy options, describe the TB prevention impact and cost-effectiveness of public health investments, and devise locally tailored TB policy portfolios.

We describe the development and functionality of this tool, and report projected TB outcomes for all U.S. states under a base case scenario. Using Massachusetts as a case study, we show how the tool can be used to simulate the costs, prevention impact, and cost-effectiveness of alternative prevention and treatment approaches.

## Methods

Tabby2 is a web application built using the Shiny web framework [9] in the R programming language [10], which provides an online open-access user interface to the Modelling Interventions for Tuberculosis in the United States (MITUS), a transmission-dynamic model of TB epidemiology and health services. The model is available on GitHub (https://github.com/PPML/MITUS/tree/tabby2) as a package for the R programming language (MITUS). The Tabby2 web application can be accessed online at https://ppmltools.org/tabby2/.

### Software architecture

The Tabby2 web application makes use of modern software engineering practices including modular design and R package-based automated testing and documentation. Tabby2 uses R packages including Shiny, Rcpp, ggplot2, dplyr, and others to run model simulations, plot outcomes, and format data for display [9–13]. The result is an online open-access web application that allows users to describe, visualize, and export modeled TB control strategy scenarios and their associated costs.

### Mathematical model

The epidemiological estimates provided by Tabby2 are generated by the MITUS package, which extends a published mathematical model of TB [14] to include risk strata that can be matched to the features of a target population by state.

**Fig. 1:**
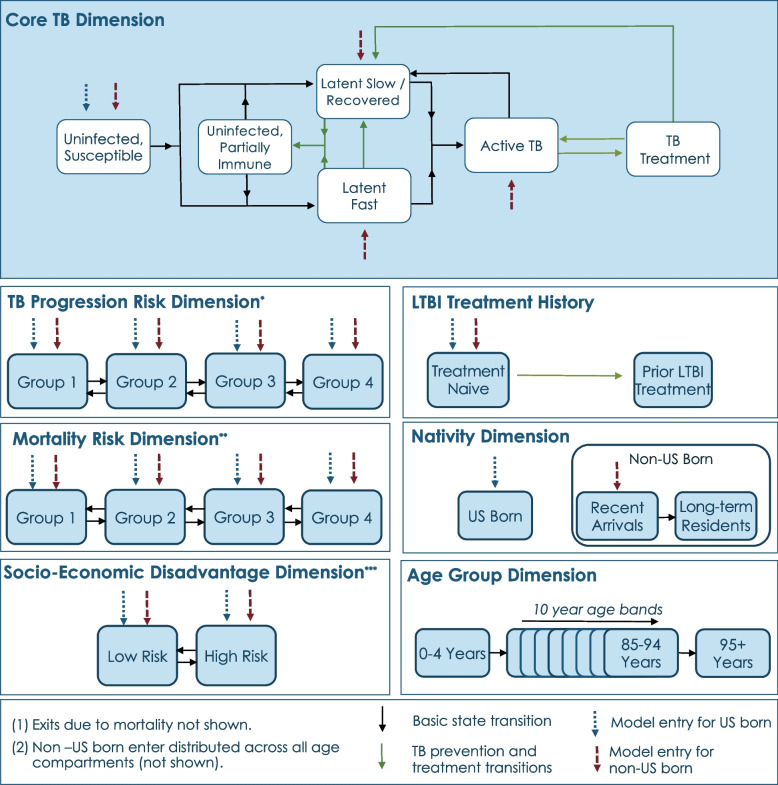
Schematic of the structure of the transmission-dynamic TB model, showing model compartments and transitions Legend: *The TB progression risk dimension represents differences in LTBI reactivation rates within the modeled population. **The mortality risk dimension represents differences in non-TB mortality rates within the modeled population. ***The socio-economic disadvantage dimension represents poor and marginalized individuals operationalized as elevated TB contact rates, elevated mortality rates, higher LTBI screening rates, and higher TB treatment default rates. We modeled TB transmission assuming assortative mixing within U.S.–born and non-U.S.–born groups, and within levels of the socio-economic disadvantage dimension (additional details described in [14])

In the MITUS model, a core TB dimension captures TB transmission, natural history, and treatment (Fig. 1). Additional dimensions represent (1) TB progression risk, (2) mortality risk, (3) socio-economic disadvantage, (4) LTBI treatment history, (5) nativity (U.S.–born or non-U.S.–born), and (6) age-based differences in disease mechanisms and risk factor prevalence. The full list of compartments represented in the model is the result of all possible combinations of these seven dimensions. The population is represented as a distribution across these dimensions, which changes over time according to entry and exit rates from each of the compartments. The model simulates historical (1950–2020) as well as current and projected (2021-2050) demography and TB epidemiology for each of the 50 U.S. states, the District of Columbia, and the United States as a whole. Previous versions of this model have been used to forecast long-term trends in TB outcomes in the United States [14] and in California [15], investigate the impact of international TB control on TB in the U.S. [16], and describe historical patterns of LTBI epidemiology [17].

### Data sources

**Fig. 2:**
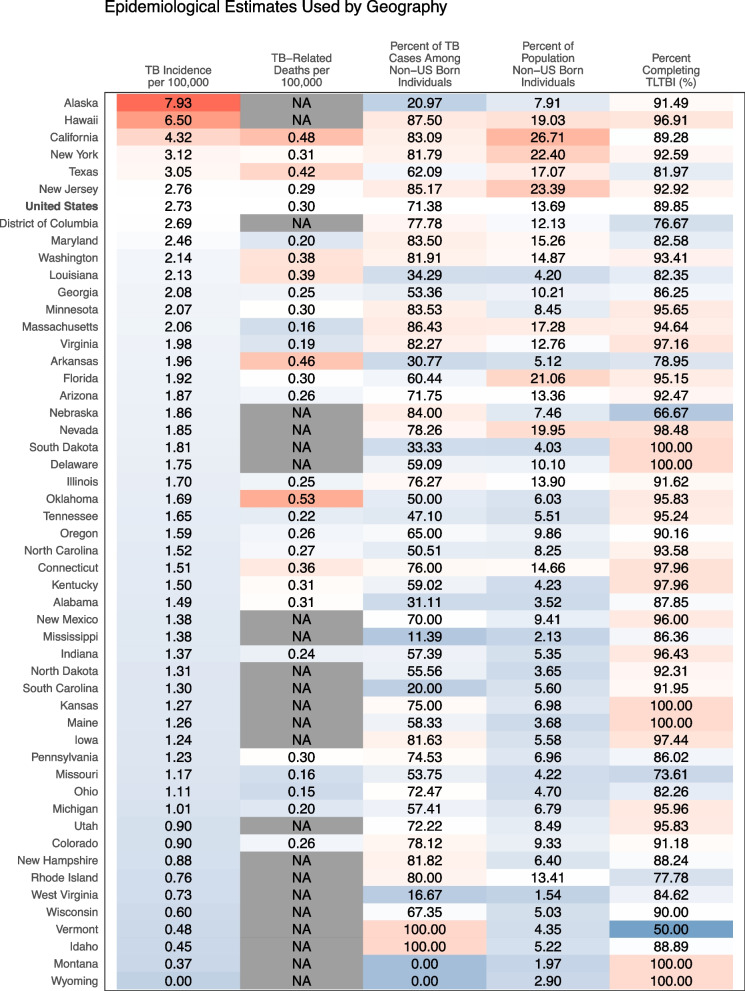
2019 Reported TB data for each U.S. state and the District of Columbia. Legend: These data were used for calibrating the underlying TB model. TB case data from National Tuberculosis Surveillance System for 2019 [6]. Deaths with TB from CDC Multiple Cause of Death for 2019 [20]. NA represents death counts under 10 which are suppressed. Population fractions from the 2019 American Community Survey [19]. Percent completing TLTBI are the 2017 values reported in the 2019 City and State Indicators Report [23]

The MITUS model incorporates a range of state-specific epidemiological and demographic data, including National Tuberculosis Surveillance System data on the distribution and trends in TB disease incidence [18], American Community Survey population estimates [19], National Center for Health Statistics data and estimates of historical and future mortality rates [20], and 2011 National Health and Nutrition Examination Survey estimates of LTBI test positivity [21]. Trends in future immigration volume are based on U.S. Census Bureau projections [22], and TB infection prevalence among future migrants is assumed to fall at 2.0% per year (details in [14]). Assumptions about the outcomes of LTBI and TB treatment are drawn from state and national reporting data on the management of contacts to TB cases [23], as well as previously published evidence [14]. Figure 2 shows key input and calibration data for the 50 modeled states and the District of Columbia.

### Model calibration

The model was fitted using a Bayesian calibration approach [24, 25]. We specified probability distributions to represent uncertainty in model parameters (priors), and likelihood functions to represent independent evidence about modeled outcomes. We then use an optimization approach combining Nelder-Mead and Broyden–Fletcher–Goldfarb–Shannon (BFGS) algorithms [26, 27] to identify the mode of the posterior distribution, calculated as the product of prior distribution and likelihood. Point estimates reported in Tabby2 represent the posterior mode of the calibrated model parameters. Additional file 1: Figure S1 shows an example of the calibration results for Massachusetts, which are also available in the online tool for each modeled geography.

To calibrate state-level models, we first constrained parameters not expected to vary between U.S. states (i.e., general features of TB natural history) to the values obtained in the national-level calibration, then calibrated the remaining parameters to state-specific epidemiological data (Additional file 1: Table S1). This two-step approach accounts for state-level variation in observed TB outcomes and risk factor distributions, while maintaining a consistent representation of TB natural history and the effect of individual risk factors.

We adjusted these calibrated models to allow for changes in TB epidemiology, mortality, and case detection during the COVID-19 pandemic. We assumed that the observed changes in TB reported cases and deaths could be attributed to four mechanisms: reduced immigration, reduced rates of diagnosis and treatment initiation for individuals with TB disease, reduced respiratory contact rates, and increased mortality among those with TB disease. Using a Bayesian optimization routine, we identified the combination of changes in these mechanisms that best reproduced 2020 TB disease case totals (overall, among recent migrants, attributed to recent transmission) and TB deaths at a national level, while maintaining consistency with reported changes in immigration and respiratory contact rates during the period [28, 29]. We applied the fitted parameter values to all states. Changes were assumed to begin in March 2020, held constant until January 2022, and returned linearly to pre-2020 values over 2022–2023.

### User interface

Tabby2 users first select a geographic area of interest on the *Introduction* page. They are then directed by the application to the *Scenarios* section, where they specify scenarios to compare on the *Predefined scenarios* and a *Build custom scenarios* pages. Next, users are guided to the *Modeled outcomes* section, where model results are available in the *Estimates*, *Time trends*, *Age groups*, *Counts of services*, and *Comparison to recent data* pages. Finally, users can use the *Economic Analysis* section to estimate the costs, health benefits, and cost-effectiveness of defined interventions in the *Input costs*, *Costs and outcomes*, and *Cost-effectiveness comparison* pages. The tool’s sidebar serves as the primary navigational aid for the user. Table 1 provides a detailed description of the functionality of each section of the webtool.

**Table 1:** Organization and function of each section of the Tabby2 user interface

Introduction	The Introduction page describes the purpose and abilities of Tabby2 and includes citations for previous work that used the model Tabby2 is based on as well details about the funding for Tabby2.
Scenarios	
*Pre-defined scenarios*	The Predefined scenarios page provides a description of the five predefined scenarios in Tabby2.
*Build custom scenarios*	The Build custom scenarios page enables Tabby2 users to create custom targeted testing and treatment, care cascade changes, or combination scenarios for simulation with Tabby2.
Modeled outcomes	
*Estimates*	On each of the Estimates, Time trends, and Age groups pages, model outcomes are visualized and available as downloads in formats including as an image in a PNG, PDF, or PPTX file, or as a data table formatted as a CSV or XLSX file.
*Time trends*	
*Age groups*	
*Counts of services*	Model informed estimates of health services are visualized as time trends and available as downloads in formats including as an image in a PNG, PDF, or PPTX file, or as a data table formatted as a CSV or XLSX file.
*Comparison to recent data*	The Comparison to recent data page allows Tabby2 users to compare Tabby2 model estimates to historically observed data.
Economic analyses	
*Cost introduction*	The Cost introduction page provides information on the economic analysis of the health interventions, default cost values, and the concepts of cost-effectiveness analysis.
*Input costs*	The Input costs page is a table of estimated average unit costs in 2020 USD. Each of these values is editable prior to submitting a costing calculation.
*Cost and outcomes*	On each of the Cost and outcomes and Cost-effectiveness pages, summarized cost and health benefits in tabular format for a specified time range.
*Cost-effectiveness*	
Further description	The Further description page of Tabby2 gives a more comprehensive description of the features of Tabby2, definitions and abbreviations used, and frequently asked questions. This page is designed to serve as a guide for new users to learn how to engage with the tool.
Changelog	The Changelog page provides the history of how Tabby2 has been updated and improved since its original release.
Feedback	The Feedback page provides users the opportunity to directly submit questions and comments to the developers of Tabby2.

### Model scenarios

In the application, a base case scenario is used to estimate future outcomes under the assumption that a similar quality and utilization level of TB services during calibration period will continue for the duration of the projection period. The application also allows users to specify alternative scenarios to compare to this base case scenario.

#### Predefined scenarios

Five scenarios are pre-specified in Tabby2 (“Predefined scenarios”): (1) provision of LTBI testing and treatment of LTBI for all new migrants entering the United States (“TLTBI for new immigrants”); (2) increased uptake of LTBI testing and treatment among high-risk populations, doubling treatment uptake within each risk group compared with current levels, and increasing the fraction cured among individuals initiating LTBI treatment, via a 3-month isoniazid-rifapentine drug regimen (“Improved TLTBI in United States”); (3) improved TB disease detection, such that the duration of untreated active disease (i.e., time from TB incidence to treatment initiation) is reduced by 50% (“Better case-detection”); (4) improved TB treatment quality, such that treatment default, failure rates, and the fraction of individuals receiving an incorrect drug regimen are reduced by 50% from current levels (“Better TB treatment”); and (5) the combination of each of these individual changes (“All improvements”). Details on the parameterization of these scenarios can be found in Additional file 1: Table S2.

#### Custom scenarios

**Fig. 3:**
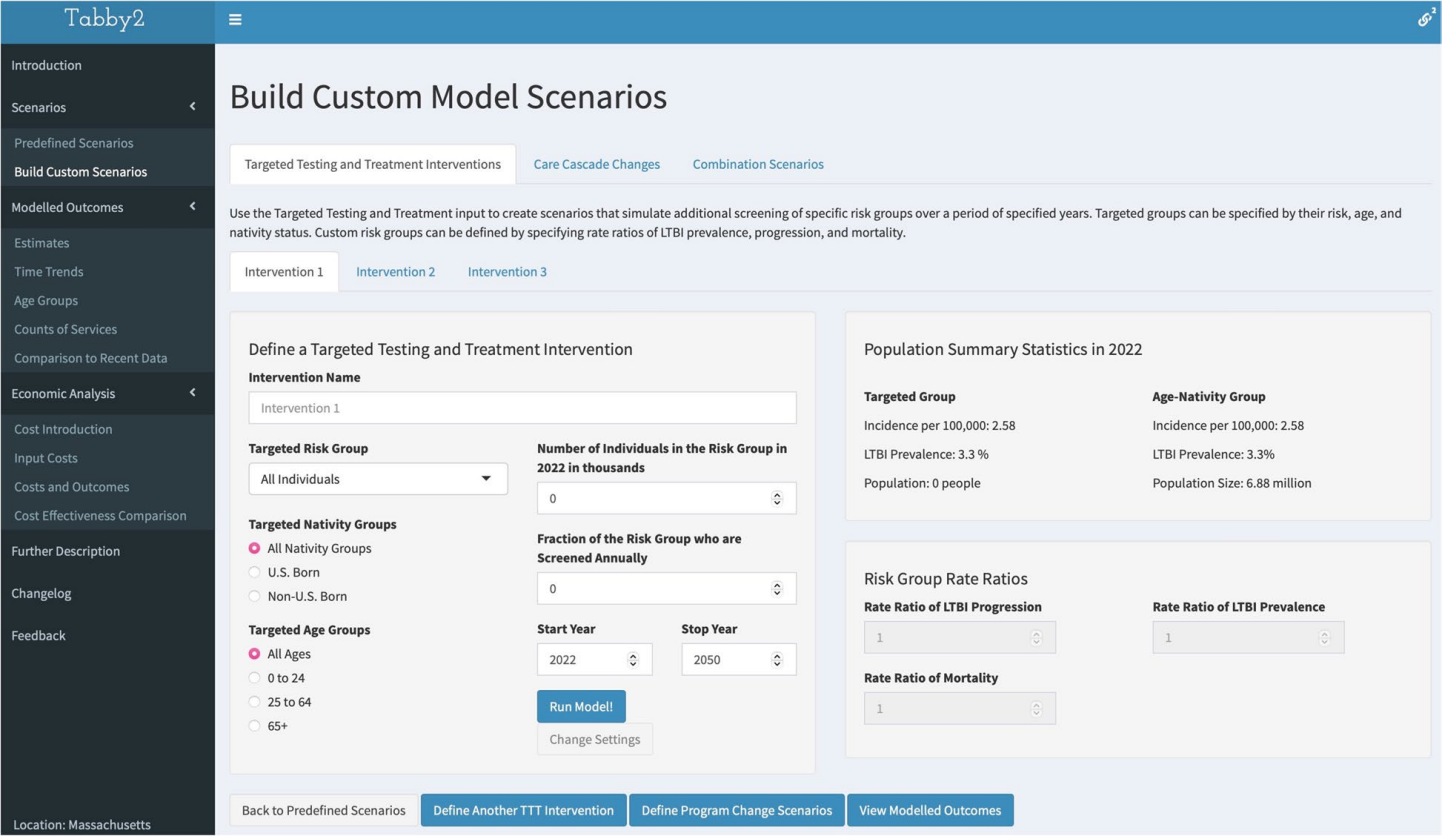
The targeted testing and treatment intervention scenario builder. Legend: Using this interface, users can design and simulate custom interventions that modify the levels of targeted testing and treatment for specific populations

In addition to the predefined scenarios, users can specify customized scenarios by modifying assumptions about the LTBI and TB care cascades, and by adding additional LTBI testing and treatment interventions for specified populations (“Custom scenarios”). Custom Scenarios allow users to select different options for Targeted Testing and Treatment of LTBI (“Targeted testing and treatment interventions”) or for TB or LTBI treatment (“Care cascade changes”). Figure 3 shows the interface of the “Targeted testing and treatment interventions” page. Users can also create scenarios that are a combination of changes, specified on the “Combination scenarios” page.

#### Health outcomes and health service utilization

For each scenario, Tabby2 reports a set of outcomes describing different features of TB epidemiology, including incident *Mycobacterium tuberculosis* infection, LTBI prevalence, TB incidence, and TB-related deaths, for the period 2022–2050. Results are presented in three interactive pages (Estimates, Time trends, Age groups) with results summarized as customizable visualizations and downloadable data tables. Results can be filtered by nativity and/or age group and presented as absolute values, percentages of the base case in the same year, or percentages of the base case in 2022. The *Counts of services* page provides time trends of the number of health services, such as LTBI tests, LTBI treatment initiations and completions, and TB disease treatment initiations and completions. An additional page of Tabby2, *Comparison to recent data*, compares modeled outcomes to recent empirical evidence for the modeled setting, to allow users to confirm the model fit.

### Economic analyses

The tool reports several outcomes related to costs and cost-effectiveness. The *Cost introduction* page provides introductory text explaining the economic analyses, and the *Input costs* page allows users to input cost data, select the analysis period, and choose whether to apply a discount rate to future costs and outcomes, which is recommended for incremental cost-effectiveness ratio calculations. Default cost inputs are based on national-level allowable Medicare reimbursements for tests and services reported by the Centers for Medicare and Medicaid [30, 31], and cost analyses conducted by the CDC [32]. Users may replace default values with state-specific inputs when these are available. All cost inputs are assumed to be in 2020 dollars. Based on these inputs, the *Costs and outcomes* page reports health outcomes and costs for each modeled scenario. The *Cost-effectiveness comparison* page provides cost-effectiveness ratios for TB cases and deaths averted, life years saved, and quality-adjusted life years (QALYs) saved for each scenario selected by the user. See Additional file 2 for a detailed list of cost inputs and economic analysis methods used on these pages of Tabby2 [30–45].

### Massachusetts case study

We used Massachusetts as a case study to demonstrate Tabby2’s functionality to investigate an intervention scenario to accelerate TB prevention through greater testing and treatment of LTBI compared to the base case scenario. Using the Custom Scenario Builder, we specified a scenario representing LTBI testing and treatment for 10% of the state’s total non-U.S.–born population annually between 2025 and 2029 (approximately 50% cumulative); all other parameters were held at base case levels. Intervention costs were calculated using national average health service and productivity costs (see Additional file 2 for values).

## Results

### Future state-level TB trends

**Fig. 4:**
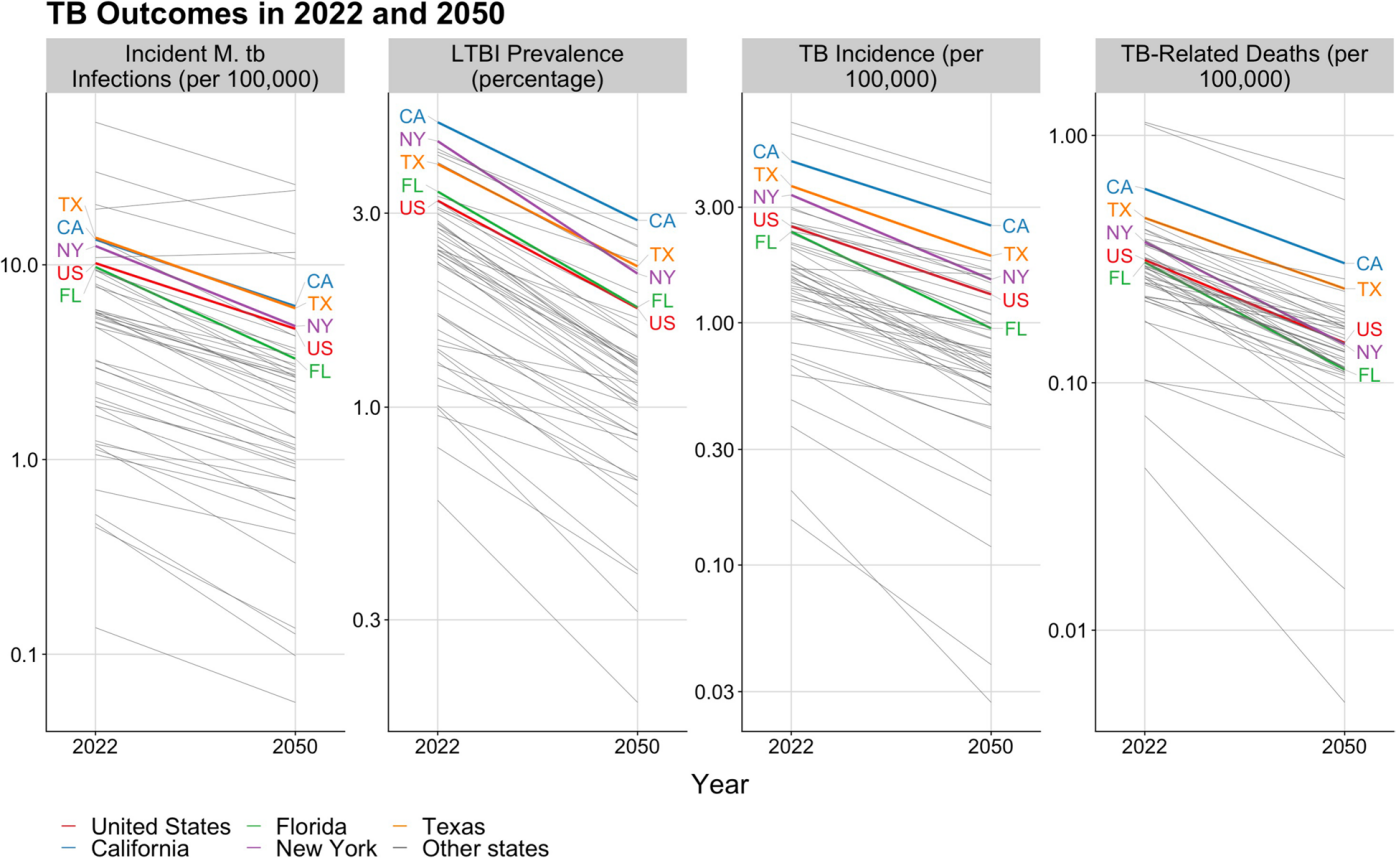
Projected trends in TB outcomes for each modeled geography, 2022 to 2050. Legend: Trends are shown on the log scale. Highlighted results represent the U.S. overall, and the four states representing over 50% of reported TB in 2019 (California, Texas, Florida, and New York)

Figure 4 reports state-level trends in TB incidence, TB-related mortality, and LTBI prevalence between 2022 and 2050 under the base case scenario. Over this period, base case TB incidence rates were projected to decline in all states. The reduction in TB incidence rates (compared to 2022 values) ranged from 20.56% in Iowa to 86.70% in Montana (48.50% median reduction across all 50 states and the District of Columbia). By 2050, TB incidence rates were projected to range from 3.77 per 100,000 people in Hawaii to 0.03 per 100,000 people in Montana (median = 0.95 per 100,000 people). In general, the rate of decline in TB incidence was projected to be higher in states with relatively low annual immigration as a fraction of total population. Across all states, base case incident TB infections, LTBI prevalence, and TB-related deaths were projected to decline to a median value of 2.20 per 100,000 people (range of 0.06–25.80 per 100,000 people across all states), 1.21% (0.59%–2.88%), and 0.14 per 100,000 people (0.005–0.67 per 100,000 people) by 2050, respectively. Numeric outputs for each outcome and state can be found in Additional file 1: Table S3.

The projections also showed systematic changes in the distribution of TB disease in the population. In 2022, the model-estimated percentage of total individuals with TB disease among non-U.S.–born persons ranged from 5.12% in Montana to 87.17% in Rhode Island (median = 68.93%). By 2050, these fractions rose to a median 83.01% (17.42–96.73%), and 46 of the 50 U.S. states and the District of Columbia had the majority (>50%) of their TB incidence among the non-U.S.–born population. The model-estimated percentage of cases among individuals age 65 or over ranged from 13.43% in North Dakota to 46.96% in West Virginia (median = 28.39%) in 2022, which was projected to increase to between 19.19% (the District of Columbia) and 60.34% (Idaho) (median = 34.18%) by 2050.

### Massachusetts case study

**Fig. 5:**
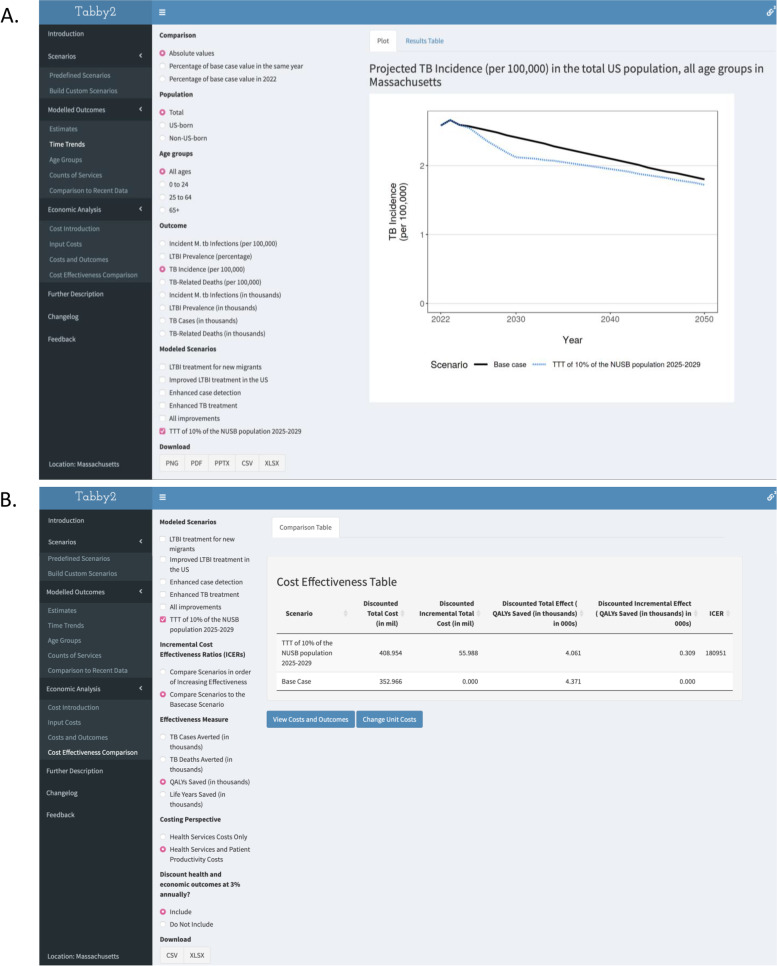
Tabby2 results pages for TB incidence trends and cost-effectiveness estimates. Legend: **A** The “Time trends” panel from Tabby2, displaying the results of the base case and expanded LTBI testing and treatment scenarios in Massachusetts. **B** The “Cost-effectiveness comparison” panel from Tabby2 displaying cost-effectiveness results for the expanded LTBI testing and treatment scenario, as compared to the base case scenario

For Massachusetts, the fraction of TB disease among non-U.S.–born individuals under the base case scenario was estimated to rise from 85.76% in 2022 to 91.49% in 2050. In this scenario, TB incidence is projected to decline from 2.57 per 100,000 people in 2025 to 1.80 per 100,000 people in 2050 (Fig. 5a). Under the improved LTBI testing and treatment intervention scenario, TB incidence falls to 1.72 per 100,000 people in 2050, a 4.65% decline.

In 2025, TB deaths in Massachusetts are estimated at 0.213 per 100,000 people and decline to 0.169 per 100,000 people under the base case, and 0.158 per 100,000 people under the intervention scenario by 2050. The results in the cost-effectiveness panel of Tabby2 are shown in Fig. 5b. Compared to the base case, the intervention scenario is estimated to avert 290 TB cases (4581 to 4290) and 34 TB-associated deaths (407 to 373) between 2022 and 2050 at an undiscounted incremental cost of 51.864 million USD. In the same timeframe, QALYs lost due to TB decreased from 8838 to 8208. From a societal perspective with 3% discounting, the incremental cost-effectiveness ratios in 2020 dollars were $286,143 per additional TB case prevented, $2,480,546 per additional death prevented, and $180,951 per additional QALY gained.

## Discussion

TB incidence rates in the United States have declined in 26 out of the last 29 years [1]. This declining trend plateaued in recent years, except for a sharp drop in 2020 associated with the COVID-19 pandemic and the subsequent increase thereafter in 2021 [5, 7]. State-level projections of TB incidence under base case assumptions created using the Tabby2 tool show ongoing declines in TB incidence rates across 50 states and the District of Columbia, consistent with other analyses [14, 46]. However, these state-level projections show considerable variation. Between 2022 and 2050, reductions in state-level incidence rates ranged from 20.56% in Iowa to 86.70% in Montana. This variation reflects inter-state differences in the drivers of TB epidemiology, including the burden of historical LTBI, access to TB prevention and treatment services, and immigration rates. TB incidence projections provide information on the progress individual states can expect with current TB control approaches based on historical parameters and provide a base case against which future progress can be compared.

The modeled base case projections also provide information about how TB disease may impact various population groups in the future. For most U.S. states and the District of Columbia, the majority of future TB disease incidence was projected to occur among the non-U.S.–born population. Over the 2022–2050 period, the projected fraction of TB cases among the non-U.S.–born population ranged from 4.9% to 86.8% across states and was generally increasing over time. This increase is a consequence of declines in TB in U.S.–born populations, immigration forecasts above historical norms, and slow reductions in TB burden among immigration cohorts [47].

The case study of expanded TB prevention services using targeted testing of 10% of non-U.S.–born individuals each year from 2025 to 2029 in Massachusetts provides an example of how alternative scenarios can be investigated via Tabby2. In this case study, expansion of LTBI testing and treatment was estimated to produce a 6% reduction in cumulative TB cases between 2025 and 2050, and an 8% reduction in TB deaths, as compared to the base case projection. Cost-effectiveness analyses for this intervention scenario produced an incremental cost-effectiveness ratio of $180,951 per QALY gained when assessed from a societal perspective compared with the base case of no additional intervention. This result is similar to results with a previous modeling study of TTT focused on the top four states by TB (California, Texas, Florida, New York), which estimated incremental cost-effectiveness ratios (ICERs) ranging from $74,000 to $174,000 (2018 USD) [48]. Other studies have shown lower costs per QALY gained from testing and treating the NUSB population [49, 50].

This case study demonstrates the potential utility of location-specific TB intervention modeling. Demand for mathematical modeling to support public health decision-making has grown in recent years. Within TB, this increase has led to the development of norms and standards for how modeling is used to support policymaking [51, 52], and the development of flexible modeling platforms that are adaptable to new settings [53, 54]. User-friendly web applications can be used to disseminate and share the results of complicated policy models, which may increase their accessibility and usefulness for policymakers. Tabby2 represents a decision-support tool that does not require significant expertise or investment in mathematical modeling and programming, is pre-calibrated to historical data from each US state and the District of Columbia and provides projections of both intervention impact and cost-effectiveness.

Several limitations accompany the requirements of an online tool. First, the tool will inherit the limitations of its underlying model, which makes simplifying assumptions about epidemiological and health service processes similar to other applied health policy models [55]. Second, the epidemiological context of modeled settings will change over time, so if not revised the model will become outdated. However, as the model is hosted online we are able to update it on a regular basis as new evidence becomes available on U.S. TB trends, migration flows, TB infection prevalence among new migrants, and intervention characteristics. Additionally, there are several states with low TB burden, which results in a fewer data to which the model can be fit, which may increase the uncertainty of the estimates for these locations. Thirdly, while the tool allows a range of different strategies to be modeled, its flexibility is limited compared with the underlying model; this design is due to the desire for a user-friendly interface. However, intervention scenarios can be added or updated according to user demand. Fourthly, as the tool expands access to new users, this audience may be less familiar with the limitations of modeled results. As such, it is critical to include clear documentation and user support to enable appropriate inputs and interpretation of results. Finally, due to the need to minimize the delay between user data entry and results being returned, the model runs one simulation based on a single best-fitting set of parameter values. In contrast, it is conventional for model-based policy analyses to run multiple simulations using a large number of plausible parameter sets in order to represent the uncertainty in modeled outcomes [56]. By basing results on a single simulation, the tool does not provide estimates of uncertainty, which may be particularly relevant for states with a small number of reported TB cases. Past analyses [14, 16], have also demonstrated the sensitivity of future TB trends to changes in migration volume and TB infection prevalence among migrants, which the user cannot vary in the current tool. To address the production limitations of Tabby2, the code for the underlying model (MITUS) is available for modifications and extensions beyond what is provided in the tool.

Despite these limitations, Tabby2 provides an accessible webtool for exploring TB epidemiology and interventions in the United States. Previous epidemiological software has required downloads which carry dependencies and compatibility requirements [53, 57]; Tabby2’s online presence only requires a modern browser, increasing access to potential users. Tabby2’s predecessor, Tabby, relied on look-up tables that limited the number of scenarios that could be viewed, and only provided estimates for the United States at a national level [58]. Tabby2’s expanded geographic focus also allows for exploration of subnational geographies based on local data, which increases the relevance of its estimates. As the model is hosted on a server and accessed through a web interface allows the model to be revised as new epidemiological and demographic data becomes available.

## Conclusions

While TB continues to decline in the United States, these reductions may be accelerated through effective intervention planning. Tabby2 provides an open-access online tool for estimating and visualizing future TB outcomes and their associated costs for each U.S. state and the District of Columbia. This tool allows users to define and simulate multiple scenarios representing improvements in targeted testing and treatment and care cascades for TB disease and LTBI treatment, using location-specific data. Tools like Tabby2 can facilitate the interactive investigation of policy options and provide decision-makers with a deeper understanding of the relative benefits of different approaches in their locality.

### Availability and requirements

Project name: Tabby2

Project home page: https://ppmltools.org/tabby2/

https://github.com/ppml/tabby2

Operating system(s): Platform independent

Programming language: R version 4.0.2

Other requirements: Requires a modern web browser such as Firefox, Safari, Microsoft Edge, Google Chrome, etc.

License: MIT License

### Supplementary Information

**Additional file 1: Figure S1.** Comparison of model estimates to recent demographic and epidemiological data, using Massachusetts as an example. **Table S1.** Parameters held fixed for all states. **Table S2.** Parameters used in the pre-defined scenarios. **Table S3.** Projected TB outcomes for each modelled geography in 2022 and 2050.

**Additional file 2.** Economic Analysis Methods. A. Definitions. B. Health impacts. TABLE EM1: Remaining life expectancy by age, for calculation of life-years and QALYs saved. TABLE EM2: Additional inputs for QALY calculation. C. Health services costs. TABLE EM3: Inputs for LTBI testing and treatment costs in USD 2020. TABLE EM4: Inputs for TB testing and treatment costs in USD 2020. D. Productivity costs. TABLE EM5: Inputs for productivity costs associated with LTBI treatment in USD 2020. TABLE EM6: Inputs for productivity costs associated with TB disease. E. Total cost estimates. F. Cost-effectiveness analysis.

## Data Availability

The datasets generated and/or analyzed during the current study are available in the MITUS GitHub repository at https://github.com/PPML/MITUS.

## Abbreviations

BFGS: Broyden–Fletcher–Goldfarb–Shannon
ICER: Incremental cost-effectiveness ratio
IGRA: Interferon-gamma release assay
LTBI: Latent tuberculosis infection
QALYs: Quality-adjusted life years
TB: Tuberculosis
The MITUS R package: Modelling Interventions on Tuberculosis in the United States
TTT: Targeted Testing and Treatment
U.S.: United States
