# Supplementary material for: Tabby2: a user-friendly web tool for forecasting state-level TB outcomes in the United States
Source: BMC Med. 2023 Aug 30;21:331. doi: 10.1186/s12916-023-02785-y (PMC10469407; doi:10.1186/s12916-023-02785-y)
Supplement: Supplementary file 1 — Additional file 1: Figure S1. Comparison of model estimates to recent demographic and epidemiological data, using Massachusetts as an example. Table S1. Parameters held fixed for all states. Table S2. Parameters used in the pre-defined scenarios. Table S3. Projected TB outcomes for each modelled geography in 2022 and 2050. [file 12916_2023_2785_MOESM1_ESM.docx]

Additional file 1 for Tabby2: A User-Friendly Web Tool for Forecasting State-Level TB Outcomes in the United States

**Supplemental figures and tables described in the main text**

Contents

1. Supplementary figures described in the main text
   1. Figure S1: Comparison of model estimates to recent demographic and epidemiological data, using Massachusetts as an example.
2. Supplementary tables described in the main text
   1. Table S1: Parameters held fixed for all states
   2. Table S2: Parameters used in the pre-defined scenarios
   3. Table S3: Projected TB outcomes for each modelled geography in 2022 and 2050

**Figure S1**: Comparison of model estimates to recent demographic and epidemiological data, using Massachusetts as an example.

Panel A shows the model fit to the TB disease trend for 2011-2020. Panel B shows the model fit to TB disease distribution across nativity, for 2011-2015 and 2016-2020. Panel C shows the model fit to the age distribution of TB cases over the period 2011-2020. Panel D shows the model fit to TB-related deaths over the period 2008-2018. These comparisons to reported data are available for each modelled geography in the Tabby2 tool.

Table S1: Parameters Fixed Across all States

| Group | Parameter(s) |
| --- | --- |
| TB Natural History | \| Probability of progressing from latent infection to TB disease (pfast) \| \| --- \| \| Adjustment of probability of progression based on age (ORpfast1, Orpfast2) \| \| Adjustment of probability of progression high risk population (ORpfastH) \| \| Adjustment of probability of progression based on previous TB treatment (ORpfastPI) \| \| Rate of fast progression from latent infection to TB disease (rfast) \| \| Rate of slow progression from latent infection to TB disease (rslow) \| \| Adjustment of rate of slow progression from latent infection to TB disease high risk (rslowH) \| \| Adjustment of rate of slow progression from latent infection to TB disease based on age (TunrslowAge) \| \| Rate of recovery from TB disease (rRecov) \| \| Rate of self-cure (rSlfCur) \| \| Mortality with TB disease (muIp) \| \| Adjustment of mortality with TB disease based on age (TunmuTbAg) \| |
| LTBI care cascade | \| Additional rate of testing for those at high risk of TB (rrTestHr) \| \| --- \| \| Reduced rate of testing for those with no risk factors for TB whose true LTBI status is negative (rrTestLrNoTb) \| \| Probability of a lack of reactivity to IGRA after previous treatment with LTBI; allows for a small proportion of population to experience an immunosenescent effect (pImmScen) \| \| Effectiveness of latent treatment (EffLt) \| |
| TB disease care cascade | \| Sensitivity of testing with TB disease (SensSp) \| \| --- \| \| Probability of cure with TB treatment completion (pCurPs) \| \| Adjustment of cure with treatment default (RrcurDef) \| \| TB treatment quality parameters (TxQualEarly and TunTxQual) \| \| Probability of cure with TB treatment (pCurPs) \| |
| Additional parameters | \| Additional mortality for high-risk populations (RRmuHR) \| \| --- \| |

Table S2: Parameterization of Pre-Defined Scenarios

| Scenario | Description / Parameterization at U.S. geographic level |
| --- | --- |
| LTBI treatment of new migrants | Test and treat all new migrants entering the United States. Without this intervention approximately 0.04% of immigrants enter with TB disease and 13.0% with LTBI. These percentages drop to 0.02% and 7.4%, respectively, with intervention. |
| Improved LTBI treatment in the United States | Double LTBI testing rate in populations at high risk for TB from 0.0031 to 0.0062.  Increase treatment initiation rates from 87.4% to 93.7% of those testing positive.  Increase treatment completion rates from 93% to 96.5% of those initiating LTBI treatment.  Increase the fraction of testing performed using IGRA from 50% to 100%.  Lower the probability of LTBI treatment toxicity from 0.003 to 0. |
| Enhanced case detection | Reduce base time to diagnosis from 3.1 months to 1.5 months for general population; 5.9 months to 2.9 months for populations at high risk for TB.  [Operationalized through increasing diagnosis of TB disease from 0.32 to 0.64.] |
| Enhanced TB treatment | Lower fraction discontinuing TB disease treatment before completion from 0.0072 to 0.0036.  Increase TB disease treatment quality from 0.94 to 0.97. |
| All Interventions | All above changes. |

* All reported changes in parameters are between 2022 and 2050.

** The parameters in these scenarios hold different values for different localities. The values presented are at the national level.

Table S3: Projected TB outcomes for each modelled geography, 2022 and 2050

| Location | Incident M. tb Infections  (per 100,000 people) | | LTBI Prevalence (percentage) | | TB Incidence (per 100,000 people) | | TB-Related Deaths  (per 100,000 people) | |
| --- | --- | --- | --- | --- | --- | --- | --- | --- |
|  | 2022 | 2050 | 2022 | 2050 | 2022 | 2050 | 2022 | 2050 |
| US | 10.2 | 4.69 | 3.22 | 1.75 | 2.5 | 1.31 | 0.31 | 0.14 |
| CA | 13.5 | 6.13 | 5.02 | 2.88 | 4.65 | 2.52 | 0.61 | 0.3 |
| TX | 13.8 | 5.98 | 3.96 | 2.22 | 3.67 | 1.89 | 0.46 | 0.24 |
| NY | 12.5 | 4.84 | 4.51 | 2.13 | 3.37 | 1.51 | 0.37 | 0.14 |
| FL | 9.75 | 3.29 | 3.39 | 1.76 | 2.38 | 0.95 | 0.31 | 0.11 |
| IL | 5.32 | 2.73 | 2.76 | 1.59 | 2.14 | 1.24 | 0.25 | 0.13 |
| NJ | 5.41 | 2.64 | 4.17 | 2.5 | 2.94 | 1.65 | 0.32 | 0.16 |
| GA | 9.47 | 3.56 | 2.99 | 1.48 | 2.34 | 0.99 | 0.28 | 0.12 |
| PA | 2.51 | 1.19 | 2.38 | 1.33 | 1.29 | 0.75 | 0.25 | 0.13 |
| MD | 5.88 | 2.5 | 4 | 2.17 | 2.99 | 1.46 | 0.36 | 0.18 |
| VA | 2.98 | 1.12 | 3.07 | 1.76 | 2.11 | 1.08 | 0.26 | 0.13 |
| MA | 4.77 | 2.7 | 3.3 | 2.29 | 2.58 | 1.8 | 0.27 | 0.17 |
| NC | 5.05 | 2.2 | 2.49 | 1.31 | 1.73 | 0.86 | 0.29 | 0.14 |
| WA | 5.55 | 2.88 | 3.1 | 1.91 | 2.53 | 1.52 | 0.4 | 0.23 |
| AZ | 5.77 | 1.72 | 2.87 | 1.2 | 2.03 | 0.67 | 0.26 | 0.08 |
| OH | 1.88 | 1.07 | 1.55 | 0.85 | 1.04 | 0.72 | 0.21 | 0.12 |
| MN | 5.71 | 3.44 | 2.44 | 1.66 | 2.39 | 1.64 | 0.3 | 0.2 |
| TN | 5.68 | 2.51 | 2.43 | 1.22 | 1.52 | 0.73 | 0.28 | 0.12 |
| HI | 30 | 14.4 | 4.32 | 2.48 | 6.73 | 3.77 | 1.11 | 0.55 |
| IN | 19.3 | 24.1 | 2.44 | 1.28 | 1.67 | 1.59 | 0.38 | 0.26 |
| MI | 1.99 | 0.95 | 2.04 | 1.02 | 1.09 | 0.61 | 0.22 | 0.11 |
| LA | 9.35 | 4.32 | 2.62 | 1.49 | 2.05 | 1.09 | 0.36 | 0.19 |
| AL | 8.74 | 3 | 2.35 | 0.98 | 1.8 | 0.65 | 0.32 | 0.12 |
| SC | 5.63 | 1.95 | 2.7 | 1.26 | 1.55 | 0.6 | 0.31 | 0.12 |
| MO | 2.95 | 1.29 | 2.25 | 1.16 | 1.23 | 0.66 | 0.22 | 0.1 |
| OR | 5.1 | 2.28 | 1.94 | 1.03 | 1.61 | 0.86 | 0.32 | 0.16 |
| MS | 7.98 | 2.81 | 2.51 | 1.03 | 1.66 | 0.61 | 0.38 | 0.14 |
| AR | 20.4 | 10.7 | 2.84 | 1.27 | 2.49 | 1.31 | 0.45 | 0.19 |
| OK | 13.6 | 4.29 | 2.58 | 1.13 | 1.68 | 0.59 | 0.33 | 0.11 |
| NV | 4.8 | 1.29 | 2.76 | 1.26 | 1.57 | 0.46 | 0.27 | 0.07 |
| KY | 10.9 | 11.6 | 2.82 | 1.3 | 1.39 | 0.93 | 0.3 | 0.15 |
| CO | 2.46 | 0.98 | 1.7 | 0.85 | 1.12 | 0.54 | 0.31 | 0.14 |
| AK | 54 | 25.8 | 4.24 | 2.73 | 6.03 | 3.39 | 1.13 | 0.67 |
| CT | 2.09 | 0.9 | 2.39 | 1.21 | 1.47 | 0.73 | 0.29 | 0.14 |
| IA | 1.25 | 0.63 | 1.42 | 1.15 | 1.25 | 0.99 | 0.22 | 0.17 |
| WI | 1.13 | 0.49 | 1.39 | 0.66 | 0.74 | 0.36 | 0.22 | 0.11 |
| NM | 3.25 | 1.14 | 2.28 | 1.08 | 1.37 | 0.53 | 0.42 | 0.19 |
| DC | 7.69 | 3.72 | 3.21 | 1.5 | 2.6 | 1.34 | 0.42 | 0.17 |
| KS | 1.69 | 0.77 | 1.68 | 0.89 | 1.06 | 0.54 | 0.25 | 0.13 |
| NE | 1.06 | 0.63 | 1.47 | 1.01 | 1.07 | 0.78 | 0.22 | 0.16 |
| DE | 9.4 | 2.65 | 1.68 | 0.78 | 1.5 | 0.52 | 0.38 | 0.11 |
| RI | 1.18 | 0.29 | 2.27 | 1.29 | 1.33 | 0.72 | 0.3 | 0.14 |
| UT | 1.88 | 0.54 | 1.01 | 0.4 | 0.67 | 0.22 | 0.18 | 0.05 |
| ID | 0.52 | 0.13 | 0.8 | 0.39 | 0.48 | 0.19 | 0.1 | 0.05 |
| ME | 1.2 | 0.78 | 1.29 | 0.61 | 0.71 | 0.37 | 0.46 | 0.24 |
| ND | 7.83 | 2.06 | 1.18 | 0.67 | 1.75 | 0.63 | 0.36 | 0.17 |
| NH | 3.2 | 1.75 | 1.13 | 0.83 | 0.83 | 0.62 | 0.25 | 0.18 |
| SD | 5.89 | 3.09 | 1.27 | 0.86 | 1.42 | 0.96 | 0.18 | 0.11 |
| WV | 0.45 | 0.14 | 1.37 | 0.57 | 0.37 | 0.12 | 0.3 | 0.09 |
| MT | 0.47 | 0.1 | 0.99 | 0.31 | 0.2 | 0.03 | 0.05 | 0.01 |
| VT | 0.7 | 0.42 | 0.95 | 0.66 | 0.61 | 0.46 | 0.1 | 0.08 |
| WY | 0.14 | 0.06 | 0.59 | 0.19 | 0.15 | 0.04 | 0.07 | 0.01 |
