# Supplementary material for: Tabby2: a user-friendly web tool for forecasting state-level TB outcomes in the United States
Source: BMC Med. 2023 Aug 30;21:331. doi: 10.1186/s12916-023-02785-y (PMC10469407; doi:10.1186/s12916-023-02785-y)
Supplement: Supplementary file 2 — Additional file 2. Economic Analysis Methods. A. Definitions. B. Health impacts. TABLE EM1: Remaining life expectancy by age, for calculation of life-years and QALYs saved. TABLE EM2: Additional inputs for QALY calculation. C. Health services costs. TABLE EM3: Inputs for LTBI testing and treatment costs in USD 2020. TABLE EM4: Inputs for TB testing and treatment costs in USD 2020. D. Productivity costs. TABLE EM5: Inputs for productivity costs associated with LTBI treatment in USD 2020. TABLE EM6: Inputs for productivity costs associated with TB disease. E. Total cost estimates. F. Cost-effectiveness analysis. [file 12916_2023_2785_MOESM2_ESM.docx]

Additional file 2 for Tabby2: A User-Friendly Web Tool for Forecasting State-Level TB Outcomes in the United States

**Economic Analysis Methods**

Contents:

1. Definitions
2. Health impacts
   1. TABLE EM1: Remaining life expectancy by age, for calculation of life-years and QALYs saved.
   2. TABLE EM2: Additional inputs for QALY calculation.
3. Health services costs
   1. TABLE EM3: Inputs for LTBI testing and treatment costs in USD 2020.
   2. TABLE EM4: Inputs for TB testing and treatment costs in USD 2020.
4. Productivity costs
   1. TABLE EM5: Inputs for productivity costs associated with LTBI treatment in USD 2020.
   2. TABLE EM6: Inputs for productivity costs associated with TB disease
5. Total cost estimates
6. Cost-effectiveness analysis

**Definitions**

**Analytical horizon**

The analytical horizon of an economic evaluation determines for which years to accrue cost and benefits included in the analysis.

**Analytical or costing perspective**

The analytical perspective of an economic evaluation determines which costs and health benefits are included in the analysis.

**CMS – Centers for Medicare & Medicaid Services**

A federal agency within the United States Department of Health and Human Services which administers the Medicare program and assists states in administration of Medicaid.

**CPT—Current Procedural Terminology**

Current procedural terminology codes are numeric keys assigned to services that a physician provides for a patient. These codes facilitate insurance reimbursements.

**Discount rate**

Potential costs and benefits occur in at different times; in order to account for these differences, a discount rate is applied to convert these to present-day values. Discounting is separate from the adjustment for inflation and is applied after adjustment for inflation.

**Disutility**

The decrement in utility (valued quality of life) due to a particular symptom or complication.

**Dominated**

When a strategy is cost-increasing and benefit-reducing relative to other strategies it is considered dominated by other strategies.

**ICER – Incremental Cost Effectiveness Ratio**

The ICER compares the incremental cost and benefit of an intervention strategy relative to the next least beneficial scenario. The ICER does not compare dominated or weakly dominated strategies.

**MDR-TB – Multidrug-resistant tuberculosis**

Multidrug-resistant tuberculosis includes strains of TB that are resistant to at least isoniazid and rifampin, two common and potent TB drugs.

**PCE Price Index -Personal Consumption Expenditure Price Index**

A United States-wide measure of prices paid for goods and services. The measure is used for describing and adjusting for inflation.

**QALY – Quality Adjusted Life Year**

A summary measure of health attainment, which considers both the quality and the quantity of life lived. One QALY is equivalent to a year of life lived in full health.

**Weakly Dominated**

Also known as extended dominance. A strategy is said to be weakly dominated if it is dominated by a linear combination of two other strategies.

**XDR-TB – Extensively drug-resistant tuberculosis**

The model uses a pre-2022 definition of extensively drug-resistant tuberculosis, which includes rare strains of TB that are resistant to isoniazid and rifampin, plus any fluoroquinolone and at least one of three injectable second-line drugs (i.e., amikacin, capreomycin, or kanamycin). Future model changes will incorporate updated definitions of extensively drug-resistant TB (TB caused by *M. tuberculosis* strains that are resistant to isoniazid and rifampin, and are also resistant to any fluoroquinolone and at least one of the following medications: levofloxacin, moxifloxacin, bedaquiline and linezolid).

**3HP – 3 months of weekly doses of 900 mg of isoniazid and 900 mg. of rifapentine**

A preferred treatment regimen for LTBI.

**3HR – 3 months of daily doses of 300 mg. of isoniazid and 600 mg. of rifampin**

A preferred treatment regimen for LTBI.

**4R – 4 months of daily doses of 600 mg. of rifampin**

A preferred treatment regimen for LTBI.

**Health impacts**

For each scenario Tabby2 quantifies health impact as TB cases averted, TB deaths averted, life-years saved, and quality adjusted life-years (QALYs) saved. These are calculated from incremental differences in health outcomes estimated by a transmission dynamic model cumulated over an analytic horizon chosen by the user. This model expands on an earlier model of future TB epidemiology in the United States [14].

Details on the calculation of each outcome are shown below. These outcomes are shown on the *Economic Analysis* section of Tabby2, in the ‘Health Effects’ table.

TB cases averted

Total TB cases for a given scenario are calculated by summing annual TB cases from 2022 through to the end of the analytic horizon, as estimated from the transmission model. TB cases averted are calculated by subtracting the total cases in a given scenario from the total cases under a counterfactual scenario (e.g., the base-case).

TB deaths averted

Total TB deaths and TB deaths averted are calculated in the same way as for TB cases.

Life-years saved

We first calculate the total life-years lost from TB for each scenario. We do so by taking the cumulative number of TB deaths in each age group from the transmission model (between 2022 and the analytic horizon) and multiplying it by a life expectancy estimate for that age group. We then sum these totals across all age groups. Life expectancy inputs for each age group are shown in Table EM1. Estimates of life-years saved are calculated by subtracting the total life-years lost to TB in a given scenario from the total life-years lost to TB under a counterfactual scenario (e.g., the base-case).

**TABLE EM1: Remaining life expectancy by age*, for calculation of life-years and QALYs saved.**

| Age Group | Life expectancy [33] |
| --- | --- |
| 0–4 years | 78.7 |
| 5–14 years | 72.0 |
| 15–24 years | 62.2 |
| 25–34 years | 52.7 |
| 35–44 years | 43.4 |
| 45–54 years | 34.2 |
| 55–64 years | 25.7 |
| 65–74 years | 18.0 |
| 75–84 years | 11.3 |
| 85–94 years | 6.19 |
| 95 years+ | 3.18 |

**The age-weighted average of life expectancies by 5-year age groups in 2018 was calculated to produce the 10-year age bands presented above.*

QALYs saved

We first calculate the total QALYs lost to TB for each scenario. These are calculated as the sum of:

1. *QALYs lost due to TB death*: these are equal to the total life-years lost from TB, as described above.
2. *QALYs lost during TB disease and TB treatment:* these are calculated as the product of total life-years lived with TB disease, including time spent on TB treatment (estimated from the MITUS model), and the disutility of TB disease.
3. *QALYs lost during LTBI treatment:* this is calculated as the product of total individuals initiating LTBI treatment (estimated from the transmission model), probability of hepatoxicity for this group, and the estimated QALYs lost per episode of hepatoxicity.

Values and sources for QALY inputs are shown in Table EM2. Estimates of QALYs saved are calculated by subtracting the total QALYs lost to TB in a given scenario from the total QALYs lost to TB under a counterfactual scenario (e.g., the base case). QALY decrements do not include those due to long-term sequelae from TB disease.

**TABLE EM2: Additional inputs for QALY calculation.**

| Input | Value | Source |
| --- | --- | --- |
| Life expectancy at the time of TB death | *See Table 1* | [33] |
| **Utility weights** |  |  |
| LTBI treatment with no toxicity | 1 |  |
| LTBI treatment with toxicity | 0.75 | [34] |
| TB disease and treatment | 0.76 | [35] |
| **Other parameters** |  |  |
| Duration of TB disease prior to TB treatment | 2.87–5.88 months (from transmission model) |  |
| Duration of TB treatment | 9 months* |  |
| Probability of toxicity during LTBI treatment | 0.003 | [36] |

Notes: *A 6-month TB treatment regimen takes, on average, 9 months to complete due to interruptions in care.

**Health services costs**

For each scenario Tabby2 estimates health services costs related to treatment of LTBI and TB disease. These are calculated from incremental differences in health service provision estimated by the MITUS transmission dynamic model cumulated over an analytic horizon chosen by the user, and unit costs estimated for each of these services. Details on the calculation of each cost outcomes are shown below. Total cost estimates are shown on the economic evaluation section of Tabby2, in the ‘Costs’ table.

Health service costs of LTBI testing and treatment

LTBI testing and treatment costs are calculated as the sum of the costs associated with:

1. *Identifying individuals for testing*: total individuals offered testing (estimated by the MITUS model) multiplied by a unit cost per person offered testing.
2. *LTBI testing*: total individuals tested (estimated by the MITUS model) multiplied by a unit cost of diagnosis. Unit costs are estimated as a weighted average of TST and IGRA costs.
3. *Ruling out TB disease*: total individuals testing positive for LTBI (estimated by the MITUS model), multiplied by the costs of TB disease screening (estimated as the cost of two chest x-rays).
4. *LTBI treatment*: total individuals initiating LTBI treatment (estimated by the MITUS model) multiplied by a unit cost of LTBI treatment. This unit cost was calculated as the average cost of 3HP, 4R, and 3HR regimens, weighted by the distribution of regimens chosen by the user.

Values for these inputs are shown in Table EM3. Several of these values represent defaults that can be adjusted by users of the webtool. All health cost estimates taken from published sources were updated to 2020 US dollar values using Bureau of Economic Analysis Personal Consumption Expenditures (PCE) separate indices for hospital and outpatient healthcare services [37].

**TABLE EM3: Inputs for LTBI testing and treatment costs in USD 2020.**

| **Input** | **Value** | **Can be edited by user?** | **Source / comment** |
| --- | --- | --- | --- |
| Cost to identify an individual for LTBI testing | $0 | **Yes** | Default to zero |
| Cost of TST | $9.38 | **Yes** | CMS 2020, Physician Fee  CPT 86580 [31] |
| Cost of IGRA | $61.98 | **Yes** | CMS 2020, Limit (Q4)  CPT 86480 [31] |
| Fraction of LTBI tests with IGRA | 0.50 | **Yes** | Default, to be updated by user |
| **Cost of ruling out TB disease before treatment initiation** | $33.20 | **Yes** |  |
| Chest x-ray (2 view) | $33.20 | No | CMS 2020, Physician’s Fee  CPT 71046 [30] |
| **Total cost of treatment with 3HP** (self-administered SAT) | $411.87 | **Yes** |  |
| Medication costs | $133 | No | [38] |
| Cost of clinic visits | $244.77 | No | [39] See note below*^,^ ** |
| Adverse events of 3HP (medical) | $34.10 | No | [39] See note below*** |
| **Total cost of treatment with 3HR by SAT** | $354.87 | **Yes** |  |
| Medication costs | $76 | No | [38] |
| Cost of clinic visits | $244.77 | No | [39] See note below*^,^ ** |
| Adverse events of 3HR (medical) | $34.10 | No | [39] See note below*** |
| **Total cost of treatment with 4R by SAT***** | $353.61 | **Yes** |  |
| Medication costs | $78 | No | [38] |
| Cost of clinic visits | $275.61 | No | [39] See note below *^,^ ** |
| Distribution of treatment of LTBI regimens |  |  |  |
| Fraction treated receiving 3HP | 0.33 | **Yes** | Default, to be updated by user |
| Fraction treated receiving 3HR | 0.33 | **Yes** | Default, to be updated by user |
| Fraction treated receiving 4R | 0.33 | **Yes** | Default, to be updated by user |

** Values were converted from 2010 to 2020 dollars using the ratio of PCE separate indices for outpatient services for each of these years (1.10) [37].*

*** Cost of clinic visit is inclusive of supplies for the associated visits.*

**** We assume a common cost of adverse effects for both isoniazid-containing regimens (3HP and 3HR). This cost is based on hepatoxicity due to the use of isoniazid as evidenced with consideration of 3HP in Sterling et al. [36]. Given a lack of differential toxicity between the regimens, we assume common proportion of toxicity in 3HR and 3HP. We assume no toxicity from the 4R regimen, as it does not use isoniazid.*

Health service cost of TB disease treatment

TB treatment costs are calculated as the number of individuals receiving TB treatment multiplied by a fixed unit cost. Inputs used in these calculations are shown in Table EM4. Of note, the transmission model does not stratify cases by drug resistance and therefore assumes a single unit cost for TB treatment. For this reason, the disease cost averted which is associated with TB cases averted by LTBI treatment may be overestimated by 10–15%. This is because the averted cases will not include MDR-TB, even though the unit cost is calculated as a weighted average that includes MDR-TB. The average cost of TB treatment includes the cost of diagnosing TB disease.

**TABLE EM4: Inputs for TB testing and treatment costs in USD 2020.**

| Input | Value (2020 USD) | Can be edited by user? | Source / Comment |
| --- | --- | --- | --- |
| Cost to identify an individual for TB testing | $0 | **Yes** | Default to zero |
| **Average cost of TB treatment** | $22,049.75 | **Yes** | Weighted average by drug-susceptibility (shown below) |
| Direct treatment costs non-MDR TB | $21,325 | No | [32] See note below* |
| Direct treatment costs MDR TB | $182,186 | No | [32] See note below** |
| Direct treatment costs XDR TB | $567,708 | No | [32] See note below** |
| **Distribution of drug strains** |  |  |  |
| Non-MDR TB | 0.9921 | No | [40] |
| MDR TB | 0.0078 | No | [40] |
| XDR TB | 0.0001 | No | [40] |

** Direct costs of non-MDR TB are calculated as the product of the probability of hospitalization, average duration of hospitalization [41] and cost per day of hospitalization estimates [42]. An average outpatient cost is also added for the total value. The values were converted to 2020 USD from 2014 USD using the ratio of PCE outpatient indices for each of these years, 1.06 [37].*

*** Converted from 2010 to 2020 dollars using the ratio of PCE inpatient and outpatient indices for each of these years, 1.21 and 1.10, respectively [37].*

**Productivity costs**

In addition to health services costs, Tabby2 calculates the loss in economic productivity associated with receipt of TB treatment and TB disease morbidity and mortality. These are calculated from incremental differences in health outcomes and health services estimated by the MITUS transmission dynamic model accumulated over an analytic horizon chosen by the user, and age-stratified estimates of economic productivity loss. Details on the calculation of these outcomes are shown below. Estimates of total productivity losses are shown on the economic evaluation section of Tabby2, in the ‘Costs’ table.

Productivity costs of LTBI treatment

For each scenario, the productivity costs associated with LTBI treatment are calculated from the cost inputs shown in Table EM5. The adverse event costs are estimated according to the user-selected distribution of drug regimens.

**TABLE EM5: Inputs for productivity costs associated with LTBI treatment in USD 2020.**

| Input | Value (2020 USD) | Source/Comment |
| --- | --- | --- |
| Initial clinic visit | $48.01 | [38] See note below* |
| Follow-up clinic visit | $28.16 | [38] See note below* |
| Adverse events of LTBI treatment with 3HP or 3HR | $5.27 | [38] See notes below*^,^ ** |
| Adverse events of LTBI treatment with 4R | $0 | See note below *** |

** These values were converted from 2010 dollars to 2020 dollars using the ratio of Bureau of Labor Statistics Average Hourly Earnings of Production and Nonsupervisory Employees index for patient productivity (1.30) [43].*

*** We assume the patient productivity per day is $220 (2016 USD) and average hospitalization stay is 7 days [41]. The average cost of adverse events with 3HP can be calculated as the product of these values and the probability of hospitalization with 3HP toxicity. This value was then converted to 2020 dollars using the ratio of Bureau of Labor Statistics Average Hourly Earnings of Production and Nonsupervisory Employees index for patient productivity for each of these years (1.14) [43].*

**** While we allow for differential LTBI treatment regimens, which have different probabilities of adverse events, we assume a common cost of these adverse effects across 3HP and 3HR as they both include isoniazid, which has some risk of hepatotoxicity. We assume no hepatotoxicity from the 4R regimen, thus include no adverse event costs. The costs of adverse events for 3HP and 3HR have been weighted by the probability of hepatotoxicity (Table EM2), which was assumed equivalent for both 3HR and 3HP.*

Productivity costs of TB disease

The productivity costs of TB disease are calculated as the sum of productivity losses from mortality and morbidity. Mortality costs for each scenario are calculated as loss of productivity given the age-specific life expectancy at TB death and the remaining age-specific annual productivity estimates. Morbidity costs are calculated as the average time loss due to outpatient TB services and the average hospitalization cost, derived as the product of the probability of hospitalization, average duration of hospitalization, and age-specific productivity estimate. The total productivity costs of TB disease are the sum of both of these values. Values used in these calculations are shown in Table EM6.

**TABLE EM6: Inputs for productivity costs associated with TB disease**

| **Input** | **Value** | **Source** |
| --- | --- | --- |
| Probability of hospitalization with TB | 0.49 | [41] |
| Average duration of hospitalization with TB | 24 days | [39] |
| Time losses due to outpatient services | 6.8 days | [39] |
| Annual productivity by age |  | [44] |
| 15–24 years old | $20,166 |  |
| 25–34 years old | $64,686 |  |
| 35–44 years old | $87,023 |  |
| 45–54 years old | $83,354 |  |
| 55–64 years old | $67,990 |  |
| 65–74 years old | $38,504 |  |
| 75+ years old | $16,017 |  |
| Lifetime productivity by age |  | [44] |
| 0–4 years old | $1,117,558 |  |
| 5–14 years old | $1,399,870 |  |
| 15–24 years old | $1,757,978 |  |
| 25–34 years old | $1,856,808 |  |
| 35–44 years old | $1,582,474 |  |
| 45–54 years old | $1,142,626 |  |
| 55–64 years old | $675,070 |  |
| 65–74 years old | $315,914 |  |
| 75–84 years old | $150,406 |  |
| 85–94 years old | $88,059 |  |
| 95+ years old | $30,805 |  |

Note: annual and lifetime labor productivity estimates for future years are adjusted for a projected real income growth of (.5% annually).

**Total cost estimates**

Two estimates of total costs are reported:

- The *total health services cost*, which sums the costs of LTBI and TB treatment cost over the analytical time horizon chosen by the user, and
- The *total cost*, which sums health services costs and productivity costs over the analytical time horizon chosen by the user.

**Cost-effectiveness analysis**

Tabby2 presents the user with several options for changing parameters of the cost-effectiveness analysis:

- *Analytic horizon*: the time period of the analysis. This is specified on the ‘Input Costs’ page. Costs and health benefits are summed over this period for the cost-effectiveness analysis, and other tables on the page.
- *Health outcome*: the user can select one of four health outcomes (TB cases, TB deaths, QALYs, and life-years) to be used in the cost-effectiveness analysis. The details of these measures are described under the health impact section.
- *Analytical/costing perspective*: healthcare system or healthcare system plus productivity losses. The user can select whether to include or exclude productivity costs from the cost-effectiveness analysis.
- *Discount rate*: the user can select whether to conduct the cost-effectiveness analysis with undiscounted costs and health outcomes, or to discount both costs and health outcomes at a fixed annual rate of 3% [45].
- *Counterfactual*: the user can select one of two approaches for calculating cost-effectiveness ratios. In the first approach, all ACERs are calculated with respect to the base-case scenario (e.g., for a given intervention (IntA), the cost-effectiveness ratio is calculated as [Costs(IntA) − Costs(base case)] ÷ [HealthBenefit(IntA) − HealthBenefit(base case)]). In the second approach, a traditional incremental cost-effectiveness analysis is conducted, with dominated strategies excluded, strategies ordered by increasing health impact, and ICERs then calculated from the incremental costs and health benefits of each strategy compared to the next least effective.

The choice of health outcome, costing perspective, discount rate, and counterfactual are selected using radio buttons on the left panel of the tool. Results of the cost-effectiveness analysis are shown in the ‘Cost-Effectiveness Table’. Cost-effectiveness results are not shown for dominated strategies (strategies that have positive costs and negative health benefits compared to their counterfactual), and these are instead labeled as ‘dominated’.
